# The optimization of calcareous soil cation exchange capacity via the feather hydrolysate and N-P fertilizers integration

**DOI:** 10.1038/s41598-025-86941-9

**Published:** 2025-02-08

**Authors:** Doaa A. Goda, Eman H. El-Gamal, Mohamed Rashad, Yasser R. Abdel-Fattah

**Affiliations:** 1 Bioprocess Development Department, Genetic Engineering and Biotechnology Research Institute (GEBRI), City of Scientific Research and Technological Applications (SRTA-City), Universities and Research Institutes Zone, New Borg El-Arab City, Alexandria, 21934 Egypt; 2 Land and Water Technologies Department, Arid Lands Cultivation Research Institute (ALCRI), City of Scientific Research and Technological Applications (SRTA-City), New Borg El-Arab City, Alexandria, 21934 Egypt

**Keywords:** Chemical fertilizer, Hydrolyzed feather, Organic fertilizer, Plant growth parameters, Soil-CEC, Biotechnology, Microbiology

## Abstract

Adding organic amendments to agricultural systems as sustainable practices is one of the most important stages toward creating a more sustainable and environmentally friendly food system. By increasing soil fertility and nutritional content, organic fertilizers increase plant productivity. Cation Exchange Capacity (CEC) is a vital indicator of soil fertility and plant nutrient availability, which is considered one of the most significant chemical parameters that affect agricultural soil quality. The main goals of the present study were to generate protein lysate from discarded feathers (enriched in different amino acids) through solid-state fermentation using Box-Behnken design (BBD). Then, assess the efficacy of hydrolyzed feather product (as an organic amendment), time, and N-P fertilizers on soil-CEC planted with maize in calcareous soil utilizing an optimization strategy that employed the central composite design and the response surface methodology (RSM). The results revealed that the protein concentration in the customized conditions was 1173.53 µg ml^−1^. With a predicted CEC of 31.416 cmol_c_ kg^−1^, the ideal circumstances for the three variables under investigation of feather hydrolysate, time, and chemical fertilizer were 20.147 ml kg^−1^ soil, 27 days, and 42.3% of the recommended dose, respectively. Using keratin hydrolysate (20 ml kg^−1^ soil) as a soil amendment significantly improved the growth parameters of maize. The leaf surface area (SA, m^2^ gm^−1^) was increased by 695% and 37% compared to the control (without any addition) and N-P fertilizer treatments (100% of the recommended dose), respectively. Keratin hydrolysate as a sustainable production of value-added organic fertilizer applied to calcareous soil shows a synergistic effect on soil-CEC and plant growth parameters.

## Introduction

Feathers play a crucial role in the life of birds by providing insulation, supporting protection, and assisting in flying^1^. It is well-known that feathers have a high keratin content that gives strength and flexibility; feathers are necessary for birds to maintain and regulate their body temperature and perform intricate movements. Notably, the poultry industry generates a substantial volume of feathers annually and the most common keratin by-product produced worldwide is chicken feathers, more than 6 million tons are thought to be produced annually^2^ underscoring the importance of developing sustainable technologies to manage this waste product. Despite being utilized in various industries such as bedding, insulation, and fashion, a significant amount of feathers still ends up as waste. Finding innovative ways to recycle or repurpose feathers could reduce environmental impact and create new economic opportunities^3^. Various advanced technologies, like enzymatic hydrolysis or pyrolysis, have been explored to convert feathers into beneficial products, such as bio-based materials, animal feed, and biofuels. Notably, transforming feather waste into valuable products may provide a sustainable resource of plant nutrients, minimize environmental pollution, and create new economic opportunities in the poultry industry^4.5^.

Feathers can be processed into feather meal or hydrolyzed feather protein, which breaks down the feather proteins into an available form of nutrients for plants. However, feather hydrolysate can be used as a slow-release organic fertilizer, especially nitrogen, providing an organic source for nitrogen fertilizer. For instance, feather meal is produced through hydrothermal processing by disrupting the structure of the feathers and is marketed as a possible N-rich supplement fertilizer^6.7^. Microbial processing is a well-suited approach for managing feathers, and the produced protein hydrolysate may have intriguing applications in agriculture^3^. Feather hydrolysate (FH) presents a more sustainable option compared to feather meal (FM), boasting a higher concentration of amino acids and nutrients and making it a valuable source of organic nitrogen fertilizer. Moreover, FH utilization as a fertilizer promotes soil health and plant growth. This innovative approach to waste management aligns with the principles of the circular economy and sustainable agriculture, creating a win-win situation for both environmental and agricultural fields^8^.

In addition to providing essential nutrients to plants, organic fertilizers can improve soil bio-physicochemical characterizations, including soil structure, water retention microbial activity, soil-pH and nutrients availability^9^. Thus, the enriched organic waste recycle and management can effectively be managed and repurposed as a promising resource for nutrient recycling, stimulating plant growth, and enhancing crop growth performance^10^. Furthermore, organic fertilizers may yield lower agronomic inputs compared to conventional mineral fertilizers, reduce the risks of chemical runoff into water resources, reduce greenhouse gas emissions, protect the environment, and promote sustainable agricultural practices^11,12^. Overall, incorporating organic fertilizers into farming practices can have numerous benefits for both farmers and the ecosystem^11,12^.This approach aligns with the principles of sustainable agriculture and circular economy, where resources are reused and recycled to create a more efficient and environmentally friendly system, further promoting ecological balance and biodiversity in our food production systems^13,14^.

**Fig. 1:**
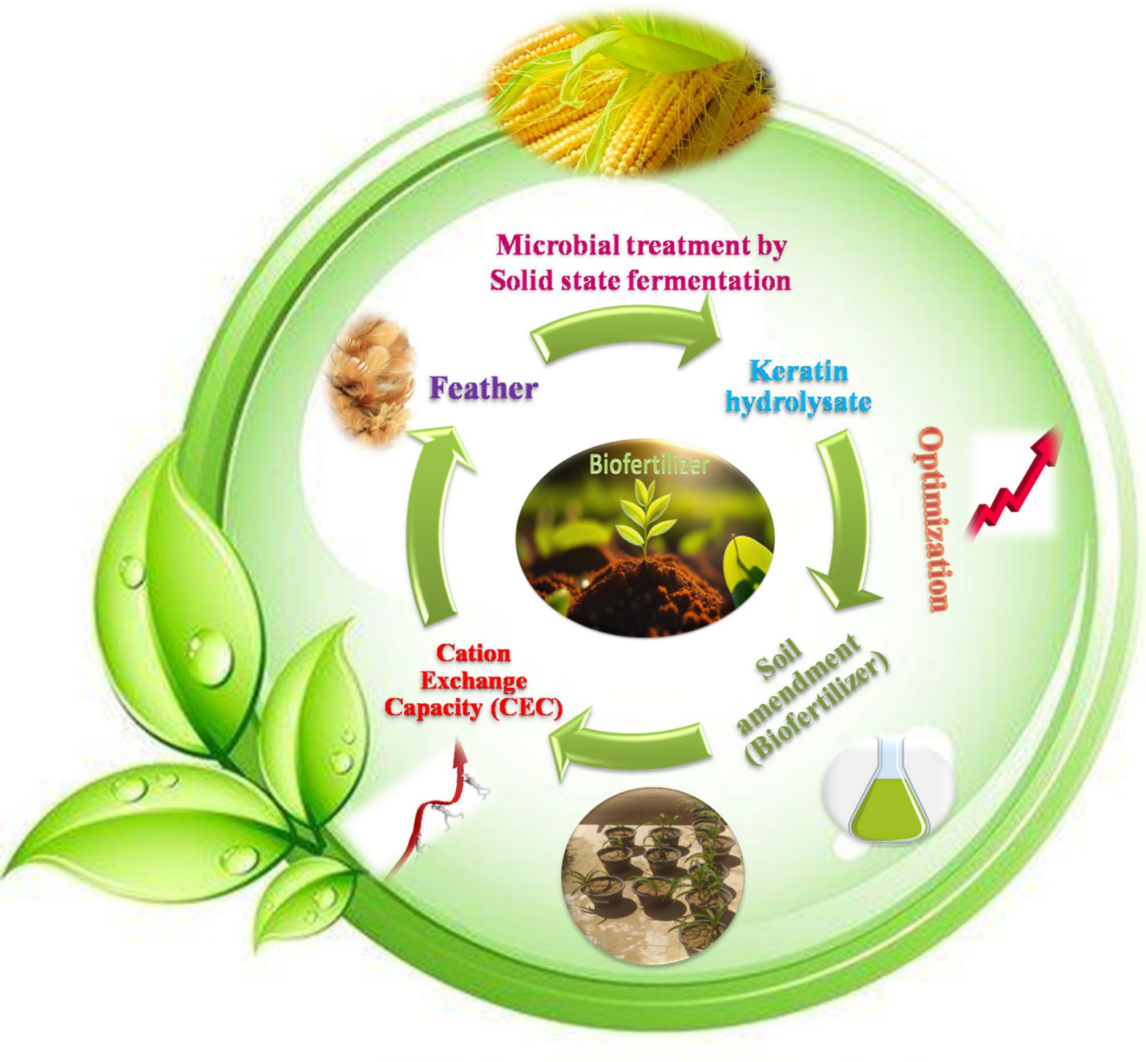
Schematic diagram illustrating protein lysate production by solid-state fermentation from discarded feathers and assess the efficacy of the produced organic fertilizer on the Cation Exchange Capacity (CEC) of amended calcareous soil through an optimization strategy utilizing the response surface methodology (RSM) by employing the central composite design.

Although the technological development of organic waste management, particularly the management of agro-industrial waste, is progressing; the practical application and evaluation of fertilizers made from the microbiological biodegradation of poultry feather waste are still in practice. The application of keratin hydrolysates for soil and plant fertilization has little data in the literature^15^. By utilizing this alternative form of feather-based fertilizer, we can maximize the nutrient content of feathers while also reducing their environmental impact through sustainable recycling practices. One environmentally acceptable method of recovering keratin waste is feather breakdown by bacteria that produce keratinase^16^.Therefore, the objectives of this study were to produce protein lysate by solid-state fermentation from discarded feathers enriched in various amino acids and assess the efficacy of the produced organic fertilizer on the Cation Exchange Capacity (CEC) of amended calcareous soil through an optimization strategy utilizing the response surface methodology (RSM) by employing the central composite design. Figure 1 provided a summary and full illustration of these steps.

## Materials and methods

### Feathers: collection and preparation

Feather waste was collected from local slaughter house situated in New Borg El-Arab City, Alexandria Governorate, Egypt. The feathers underwent a thorough cleaning process by washing detergent, followed by extreme rinsing with tap and distilled water to ensure that feathers were effectively free from contaminants such as oils, dirt, or other impurities. Subsequently, they were dried for six hours at 50 °C^17,18^. Once cleaned and properly prepared, the feathers underwent enzymatic degradation. This process is essential for effectively converting feather waste into valuable products.

### Preparing the sample for inoculation

After the 48-hour incubation period at 45 °C and 200 rpm, the culture from *Laceyella sacchari* YNDH was visually inspected for growth and turbidity. Once it was confirmed that the culture had reached the desired density, it was ready to be used as an inoculum for further experiments or bioprocesses.

### Enhancing feather protein lysate through solid state fermentation (SSF) for the production of organic fertilizer

Solid state fermentation was used to enhance the nutritional content of feathers, which primarily consists of soluble protein, using response surface methodology-based optimization process (Box-Behnken design, BBD). The primary goal of the BBD is thought to be an improvement in the protein content of the lysate. As demonstrated in Table 1, three factors (feather conc. (A), moisture % (B), and time (C)) were examined at three different levels (high, medium, and low), denoted by the levels + 1, 0 and − 1, respectively.

**Table 1:** Statistical analysis of BB design showing matrix for the selected 3-variables influencing solid state fermentation of chicken feather for protein lysate production.

Trial	Variables			Protein conc. (µg/ml)		
	A Feather	B Moisture	C Time	Experimental	Predicted	Residual
1	– 1	– 1	0	155.88	143.38	12.50
2	1	– 1	0	608.82	675.74	– 66.91
3	– 1	1	0	502.94	436.03	66.91
4	1	1	0	920.59	933.09	– 12.50
5	– 1	0	– 1	605.88	594.12	11.76
6	1	0	– 1	1173.53	1082.35	91.18
7	– 1	0	1	394.12	485.29	– 91.18
8	1	0	1	1014.71	1026.47	– 11.76
9	0	– 1	– 1	361.76	386.03	– 24.26
10	0	1	– 1	547.06	625.74	– 78.68
11	0	– 1	1	347.06	268.38	78.68
12	0	1	1	602.94	578.68	24.26
13	0	0	0	629.41	644.12	– 14.71
14	0	0	0	658.82	644.12	14.71

Variables	Code	Coded level and actual level				
		– 1	0	1		
Feather (g)	A	1	2	3		
Moisture (%)	B	60	70	80		
Time (day)	C	3	4	5		

Fit statistics			
Std. dev.	100.20	R²	0.9590
Mean	608.82	Adjusted R²	0.8668
C.V. %	16.46	Adeq precision	11.0879

The nutrient solution was carefully prepared to provide the necessary elements for the growth and development of the microorganisms in the feather-supplemented (SSF) medium according to^19^ as the following (%w/v): yeast extract, 0.04; NH_4_Cl, 0.08; K_2_HPO_4_, 0.01; KH_2_PO_4_, 0.01; NaNO_3_, 0.016; MgSO_4_ 7H_2_O, 0.02) at pH 8; inoculum size, 2%; and cultivation temperature, 45 °C.

Following fermentation, the flasks were shaken in a rotary shaker at 150 rpm to extract the protein hydrolysate using deionized water. After that, the fermented material was extracted from the hydrolysate using filtration (Whitman No. 1 paper), and the filtrate was then spun for 10 min at 7,000 rpm. For the next investigation, the supernatant served as a source of protein hydrolysate^20^. By employing the bovine serum albumin standard curve, the Lowry method was utilized for calculating the total soluble protein content^21,22^.

### Approach for statistical optimization of CEC level

#### Central composite design (CCD)

CCD was used to characterize the response surface’s characteristics in the experimental area, assess the optimal values of the important factors, and determine how interactions among the chosen variables (A: feather hydrolysate as organic fertilizer; B: time; C: N-P chemical fertilizer) affect the CEC level of calcareous soil planted with maize. The polynomial model was fitted using the five-level, three-factorial CCD approach, and the experimental errors were computed using the center point’s standard deviation.

Five levels of analysis were conducted for each of the independent variables: − α, − 1, 0, + 1, and + α, using α = 1.681738. Every trial was carried out twice, and the responses were determined by taking the mean of the two sets of results (CEC level). Table 2 provides an overview of the research methodology matrix’s form as well as the coded and real levels of the chosen variables under examination.

**Table 2:** BB designed matrix for the selected 3-variables influencing soil-CEC central composite.

Trial	Variables				CEC (cmol_c_ kg^−1^)		
	A Organic amendment	B Time	C Chemical fertilizer (*N*-*P*)		Experimental	Predicted	Residual
1	1	– 1	1		24.63	24.96	– 0.332
2	0	0	0		24.57	24.03	0.537
3	1	1	– 1		27.95	27.49	0.457
4	– 1	1	1		21.23	19.87	1.366
5	0	0	0		24.61	24.03	0.582
6	– 1	– 1	– 1		20.86	20.51	0.348
7	– 1	– 1	1		21.70	21.32	0.383
8	– 1	1	– 1		20.34	19.17	1.173
9	0	0	0		23.40	24.03	– 0.633
10	1	– 1	– 1		27.33	27.86	– 0.525
11	0	0	0		24.62	24.03	0.586
12	1	1	1		24.97	24.48	0.492
13	0	0	– 1.6329		21.76	22.25	– 0.491
14	0	– 1.6329	0		24.06	23.58	0.475
15	1.6329	0	0		30.93	30.59	0.342
16	0	0	0		23.67	24.03	– 0.362
17	0	1.6329	0		20.36	22.09	– 1.737
18	– 1.6329	0	0		19.22	20.82	– 1.604
19	0	0	0		23.74	24.03	– 0.289
20	0		0	1.63296	19.68	20.45	– 0.770

Variables	Code	Coded level and actual level				
		– 1.6329	– 1	0	1	1.6329
Organic amendment (ml/5kg soil)	A	35	50	75	100	115
Time (day)	B	15.8	21	35	49	57.8
Chemical fertilizer (N, P) (%)	C	27.4	40	60	80	92.6

A series of the three independent selected variables were tested in twenty trials in accordance with the corresponding CCD design. In order to identify the primary and secondary effects of the independent components in accordance with the response value maximization with the ideal level for every variable, statistical analysis was done using quadratic regression. Factors as a function of response values can be expressed by the quadratic equation below:

Y = β0 + Σi βi Xi + Σij βijXiXj + Σii βii X2i

where the cross-product and quadratic coefficients are denoted by βij and βii, respectively, and the independent variables are represented by Xi and Xj. To assess the equation model and make certain that the estimated values of every variable were derived correctly, laboratory validation was carried out.

#### Pot experimental setup and sampling

A surface calcareous sandy-loam soil (top 30 cm depth) was collected from City of Scientific Research and Technological Applications (SRTA-City) Experimental Farm located at Old Borg Al-Arab City (30° 53′ 33.17″ N, 29° 22′ 46.43″ E), West Alexandria, Egypt. The main chemical properties of collected soil according to the method described in Page et al.^23^ are presented in Table 3. The pH of the soil was evaluated in 1:2.5 (w/v) soil-water suspensions after 30 min of shaking, the pH was determined using a pH meter, and the saturated soil paste extract’s electric conductivity (EC, dS m^−1^) was measured. Both parameters were measured by a Multi-Parameter pH-ORP-Conductivity-TDS-TEMP Bench Meter (Adwa, AD8000 Professional). The available nitrogen forms (avail. NH_4_^+^ and avail. NO_3_^−^) were extracted by KCl solution (2.0 M) and ascertained using the 30s Vapodest Gerhardt Kjeldahel distillation unit^24^. The accessible phosphorus form in the soil was extracted using 0.5 M NaHCO_3_ (pH 8.5) and identified at 882 wavelength based on blue color density (the ascorbic acid technique) using a PG Instruments T80 UV/VIS Spectrophotometer^25^. The available potassium form in soil was extracted by a 1:10 soil suspension of neutral ammonium acetate solution (1 N) and measured by Atomic Absorption Spectrometry (AAS), Analytik Jena GmbH - ZEEnit 700, Jena, Germany^26^. Available soil micronutrients (Fe, Zn, Mn, and Cu) were determined in the clear extraction of 0.005 M diethylene triamine penta acetic acid (DTPA) according to Lindsay and Norvell^27^ and determined using ASS. By using the digested and determined Kjeldahel distillation method, total nitrogen (T.N.) was ascertained in accordance with Bremner and Mulvaney^28^. The Walkley-Black method of organic carbon (OC) was followed to determine total and dissolved organic carbon using the wet oxidation method with K_2_Cr_2_O_7_ (1 N), H_2_SO_4_, and H_3_PO_4_, and titration the excess of dichromate by ammonium ferrous sulfate^29^. Dissolved organic carbon (DOC) filtrate extraction was obtained by shaking a 1:5 (w/v) suspension at room temperature for 24 h. The ammonium-acetate compulsory (CEC-NH_4_^+^) displacement method was followed to determine the soil cation exchange capacity (CEC, cmol_c_ kg^−1^). The soil surface was saturated with 1 M sodium acetate (pH 8.2) to remove the exchangeable cations of K+, Ca^2+^, and Mg^2+^ by Na^+^ ion. Then the soil sample was washed with ethanol to eliminate the surplus sodium ions from the surface. Finally, the sodium ions were replaced by an ammonium-acetate solution (1 M, pH 7), and the released amount of sodium ions was measured by AAS. The calcimeter technique was used for estimating the total level of calcium carbonate^30^.

**Table 3:** The primary chemical properties of the collected soil.

Parameters	Unit	Value
EC	dS m^−1^	2.72
pH	(1:2.5, W: V)	8.48
NO_3_^−^	mg kg^−1^	154
NH_4_^+^	mg kg^−1^	98
P	mg kg^−1^	9
K	mg kg^−1^	485
Cu^2+^	mg kg^−1^	0.93
Fe^2+^	mg kg^−1^	5.15
Zn^2+^	mg kg^−1^	2.2
Mn^2+^	mg kg^−1^	5.5
T.N.	%	0.04
O.M.	%	1.52
DOC	%	0.006
C/N	ratio	22
CEC	cmol kg^−1^	13.98
T.C.	%	32.4

*EC electric conductivity; *T.N.* total nitrogen; *O.M.* organic matter; *DOC* dissolved organic carbon; *CEC* cation exchange capacity; *T.C.* total calcium carbonate.

Notably, this soil comprised 18.7% clay, 16% silt, and 65.3% sand, with a water-holding capacity (WHC) of 50%. The soil was identified as alkaline and slightly saline (FAO, 1998) according to its values of pH (8.48) and electric conductivity (EC, 2.72 dS m^−1^). It exhibited very low total nitrogen (T.N, 0.04%), moderate organic carbon content (O.M., 1.52%), extremely low DOC not exceeds 0.006%, accepted ratio of C: N and high percentage of total calcium carbonate (T.C., 32.4%).

The collected soils were mixed and homogenized through a 5-mm sieving process. Subsequently, the soil mixture was transferred into plastic pots (25 cm diameter and 30 cm length). The soil was thoroughly treated with N-P-K fertilizers following the recommendations of the Ministry of Agriculture and Land Reclamation^31^. In all treatments, the soil was mixed, once before seeding with single superphosphate fertilizer (15.5% P_2_O_5_, 480 kg ha^−1^) and potassium sulfate (48% K_2_O, 120 kg ha^−1^). Nitrogen fertilizer (ammonium nitrate, 33.5% N, 288 kg ha^−1^) was divided into three equal doses; the first one-third was applied after seedling emergence, and the remaining two-thirds were applied 15 and 25 days later from germination. White maize grains (*Zea Mays* L., single-cross hybrids 83) were hand-sown in April 2023. The seeds were uniformly dispersed on the soil surface and then covered with soil (5 cm layer). The levels of N-P fertilizers and keratin hydrolysate from chicken feathers in different concentrations are illustrated in Table 2. The keratin treatments were mixed with the irrigated water in the first irrigation application. A period of 7 days for germination and seedling establishment was set after sowing, where irrigation was applied in equal quantity every 3–4 days. The soil samples were collected according to the factor of time (day), as illustrated in Table 2. Additionally, to evaluate the effects of feather keratin hydrolysate application on the growth parameters of maize plants and the chemical parameters of the soil after harvesting (after 60 days from germination), separate trials were carried out in triplicate: control (without fertilizer addition), 100% of the recommended chemical fertilizer and feather keratin hydrolysate in completely randomized design (CRD). Finally, plant and soil samples were analyzed from all pot treatments.

#### Soil, keratin hydrolysate, and plant analyses

The chemical parameters of soil samples during and after experiment were analyzed as described above. Feather keratin hydrolysate extraction before the experiment was analyzed (Table 4). The total amount P, K and micronutrients in keratin hydrolysate were measured in the clear solution of the wet digestion using diluted HNO_3_ and H_2_O_2_ by atomic absorption spectroscopy AAS.

**Table 4:** The chemical analysis of the keratin hydrolysates extraction.

Sample Name	Unit	Extraction
		A
pH	–	9.10
EC	dS/m	5.19
P	%	0.14
K	%	1.53
Na	%	0.14
Ca	%	1.53
Mg	%	0.29
Cu	mg L^−1^	0.95
Mn	mg L^−1^	1.99
Fe	mg L^−1^	70.58
Ni	mg L^−1^	nd

Some maize Plant growth parameters after 60 days such as plant height (cm), shoot fresh weight (g), shoot dry weight (g), leaf area per plant (cm^2^), number of leaves per plant, root fresh weight (g), root dry weight (g) were assessed. Total nutrients from ash (500 °C for 6 h) of plant samples were extracted using *Aqua regia* (1 HNO_3_: 3 HCl) and detected by AAS. Total phosphorus content in Keratin hydrolysate and plant samples were determined by ammonium para molybdate-vanadate method at 420 wave length depending on yellow color density using spectrophotometer^32^.

### Statistical techniques for analyzing data of central composite design (CCD)

The obtained data from CCD were subjected to multiple linear regression analysis using Design-Expert 13 software. The statistical significance of the components was assessed using ANOVA, and the F-value was calculated at a probability (p-value) of less than 0.05. The adjusted determination coefficient (R^2^adj.) and multiple coefficients of correlation (R^2^) were calculated to assess the efficacy of the framework. A three-dimensional diagram was created using Design-Expert 13 software to show each response’s simultaneous influence of the three most significant distinct variables. Furthermore, a statistical analysis was conducted on the data from the separate experiment of applying feather keratin hydrolysate application and N-P fertilizer relative to untreated soil was performed. The CoStat statistical analysis system (Version 6.303, CoHort, USA, 1998–2004) was used to examine the analysis of variance (ANOVA) and compare differences between the means at least significant differences (LSD, *p* < 0.05).

## Results and discussion

### Improving feather protein lysate for the creation of organic fertilizer by (SSF) using Box-Behnken design (BBD)

Instead of using the traditional optimization method to attain the ideal response region for protein concentration using feathers as a supplier of carbon, the Box-Behnken strategy was applied to optimize the creation of protein lysate and enhance its production concentration yield. Thereby, the RSM was applied to study how feather (A), moisture (B), and time (C) interact to influence protein lysate concentration (Table 1). A linear multiple regression analysis approach with fourteen trials was used to analyze the three parameters. Model fit was assessed by examining the amount of protein production determination coefficient (R^2^ = 0.9590, Table 1), which shows that only 4.1% of the responses were deemed unsuitable for the employed model.

The results showed that both the values of R^2^ and R^2^adjusted for the creation of protein lysate using feather as a carbon producer were estimated to be 0.959 and 0.8668, respectively. Regression approaches with R^2^ levels above 0.9 are considered to have an extremely high correlation, according to Cui et al.^33^. Adeq Precision calculates the signal-to-noise ratio. The ratio ought to be more than 4. The obtained ratio in this study was 11.08 indicating a strong enough signal. Few statistical evaluations were carried out to verify the methodology’s accuracy. The normal probability plot (NPP) of the residuals is a crucial graphical tool for examining the residuals’ distribution and determining the model’s appropriateness.

**Fig. 2:**
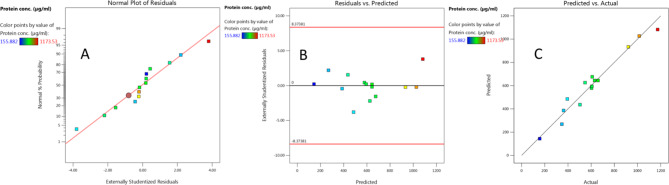
Box-Behnken design: (**A**) normal probability plot (NPP) of the residuals to assess whether the model is adequate, (**B**) internally studentized residuals versus predicted protein conc., and (**C**) plot of predicted versus actual results of protein conc.

In Fig. 2A, the model-estimated response data was displayed against the NPP of the studentized residuals. The predicted protein output is plotted versus the studentized residuals in Fig. 2B, the model’s accuracy was confirmed by the residuals’ homogeneous and irregular distribution above as well as below the zero line, which lacks any observable pattern and indicates that the residuals’ variance is constant^34^. Figure 2C, which plots predicted against real protein production and has points around the fitted line, illustrates the strong correlation between the expected results and the experimental findings of protein concentration, supporting the accuracy of the model.

**Fig. 3:**
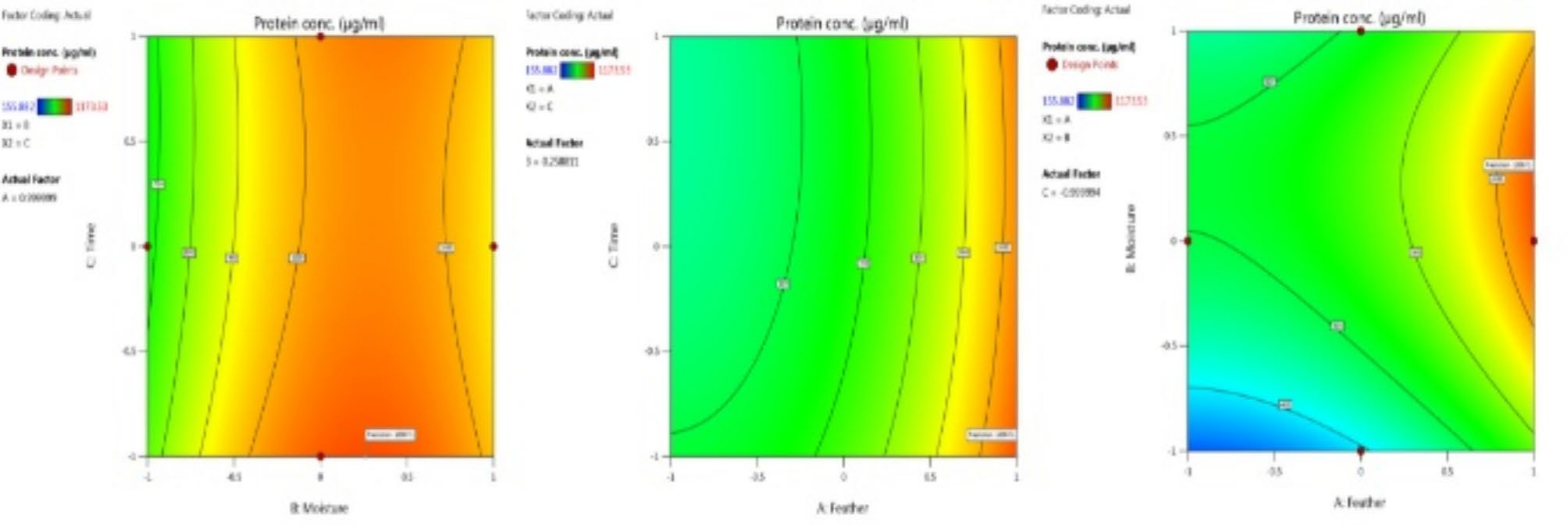
A 2D contour graphs illustrate the effects of feather (**A**), moisture (**B**), and time (**C**) on protein conc.

To ascertain the optimal concentrations for each of those factors under investigation and the consequences of how they interact on the anticipated production of protein lysate, the 2D illustrated contours were created for the pairwise combination of all three parameters (A_feather_. B_moisture_, B_moisture_. C_time_ and A_feather_. C_time_) (Fig. 3). Contour plots are a helpful visual aid for study results. They show that increased feather content, mild wetness, and shorter times produced better yields of protein lysate concentrate.

To find the ideal point of variation within the experimental restrictions, a second-order polynomial functionwas fitted to the experimental data:

Y_Protein conc_. (µg/ml) = 644.11 + 257.35A_feather_ + 137.5B_moisture_− 41.17C_time_− 0.6938A_feather_ * B_moisture_ + 13.23A_feather_ * C_time_ + 17.64 B_moisture_ * C_time_ + 117.64(A_feather_)^2^ − 214.70 (B_moisture_)^2^ + 35.294 (C_time_)^2^.

With a predicted protein concentration 1096.71 µg/ml, the maximum level of the polynomial model indicates the optimal values of the three variables under consideration: feather, 2.9 g; moisture, 72.59%; and time, 3.01 day.

An assurance experiment was conducted under ideal conditions to confirm the accuracy of the quadratic polynomial and track protein concentration in the customized conditions. Under these circumstances, an experimental protein concentration of 1173.53 µg/ml was obtained, which is within a 107% accuracy range of the value predicted by the regression model (1096.71 µg/ml), indicating the model’s validity, this aligns with the results of Senthilkumar et al.^35^, who outlined the importance and practicality of the optimization approach.

### Statistical optimization of CEC level by utilizing hydrolyzed feather and N-P fertilizers integration

RSM is a statistical empirical method that uses experimental evidence to assess a regression framework and improve a variable’s response that is influenced by several independent input factors^36^. The central-composite design approach, which is a commonly used analytical technique in RSM, was utilized to determine the best amounts of the three stated parameters (organic amendment (A), time (B), and chemical fertilizer (C)). The experimental error was computed using the six center locations. The experimental, estimated CEC level and residuals are summarized in Table 2.

The precision of the CEC model and the optimization’s dependability were evaluated through the application of statistical regression research parameters. Numerous parameters, including the determination coefficient (R^2^) value, predicted R^2^ value, modified R^2^ value, and F-value were evaluated by statistical regression analysis (Table 5). The correlation coefficient (R^2^) with the highest amount indicated the most closely connected connection among the experimental outcomes and the expected values anticipated by the selected model^37^. As R^2^ = 0.92, the model Consequently, may precisely predict the connection among the variables impacting the CEC level, as basically 92% of the variance in the CEC level could be explained by the independent variables studied. Furthermore, the high value of Fisher’s F-test (13.6) and adjusted R^2^ value (0.856) with a low p-value (0.0001), and insignificant lack of fit for CEC level productivity (F-value = 7.156; p-value = 0.0249) affirm the established model’s importance and validity.

**Table 5:** Statistical analysis of BB design showing *coefficients*,* t –*and *p-values* for significant variables affecting on CEC.

Fit statistics			
Std. dev.	1.13	R²	0.9245
Mean	23.48	Adjusted R²	0.8565
C.V. %	4.8	Predicted R²	0.4892
		Adeq precision	14.3256

ANOVA for quadratic model						Coefficients in terms of coded factors			
Source	Sum of squares	df	Mean square	F-value	*p*-value	Coefficient estimate	Standard error	95% CI high	
Model	155.66	9	17.295	13.6	0.0001	24.033	0.4585	25.055	Significant
A-organic amendment	119.29	1	119.29	93.83	2.125	2.991	0.3087	3.6791	
B-time	2.76	1	2.764	2.174	0.171	– 0.4553	0.3087	0.232	
C-chemical fertilizer(N, P)	4.032	1	4.0328	3.172	0.1052	– 0.5499	0.3087	0.138	
AB	0.4623	1	0.4693	0.3692	0.5569	0.2422	0.3986	1.1304	
AC	6.87	1	6.8731	5.406	0.0423	– 0.9269	0.3986	– 0.0386	
BC	0.0062	1	0.0064	0.005	0.9445	– 0.0284	0.3986	0.8597	
A²	5.2202	1	5.2209	4.106	0.0702	0.6287	0.3102	1.32	
B²	2.6349	1	2.6349	2.072	0.18051	– 0.4466	0.3102	0.244	
C^2^	13.337	1	13.337	10.491	0.0088	– 1.004	0.31023	– 0.313	
Residual	12.712	10	1.2712						
Lack of fit	11.154	5	2.2308	7.156	0.0249				Significant
Pure error	1.5585	5	0.3117						
Cor total	168.372	19							

**Fig. 4:**
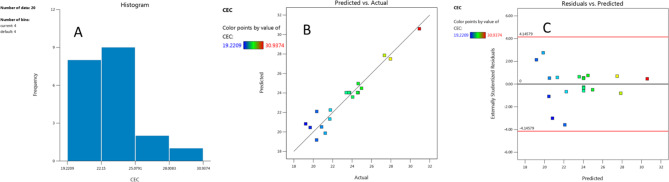
Box-Behnken design (BBD): (**A**) histogram showing a broad spectrum of CEC level ranged from 19.22 to 30.93cmol kg^−1^, (**B**) plot of predicted versus actual results of CEC level, (**C**) internally studentized residuals versus predicted CEC level.

Overall, the CEC level ranged from 19.22 to 30.93 cmol_c_ kg^−1^ as shown in histogram Fig. 4A. The CCD results were then subjected to ANOVA computations and mathematical modeling of numerous regression (Table 5). The substantial connection between the expected results and the actual CEC level findings, as demonstrated in Fig. 4B (a predicted plot against real CEC level with points nearest to the fitted line), validates the accuracy of the model. The estimated CEC level is plotted versus the studentized residuals in Fig. 4C. The model’s accuracy is validated by the residuals’ uniform and random distribution above and below the zero line, which lacks any visible pattern and indicates that the residual variance is constant.

**Fig. 5:**
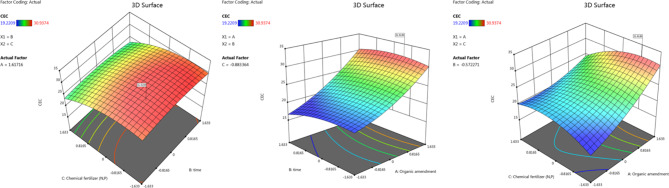
A 3D response surface and contour graphs illustrate the effects of keratin hydrolysate (**A**), time (**B**), and chemical fertilizer(**C**) on CEC.

**Fig. 6:**
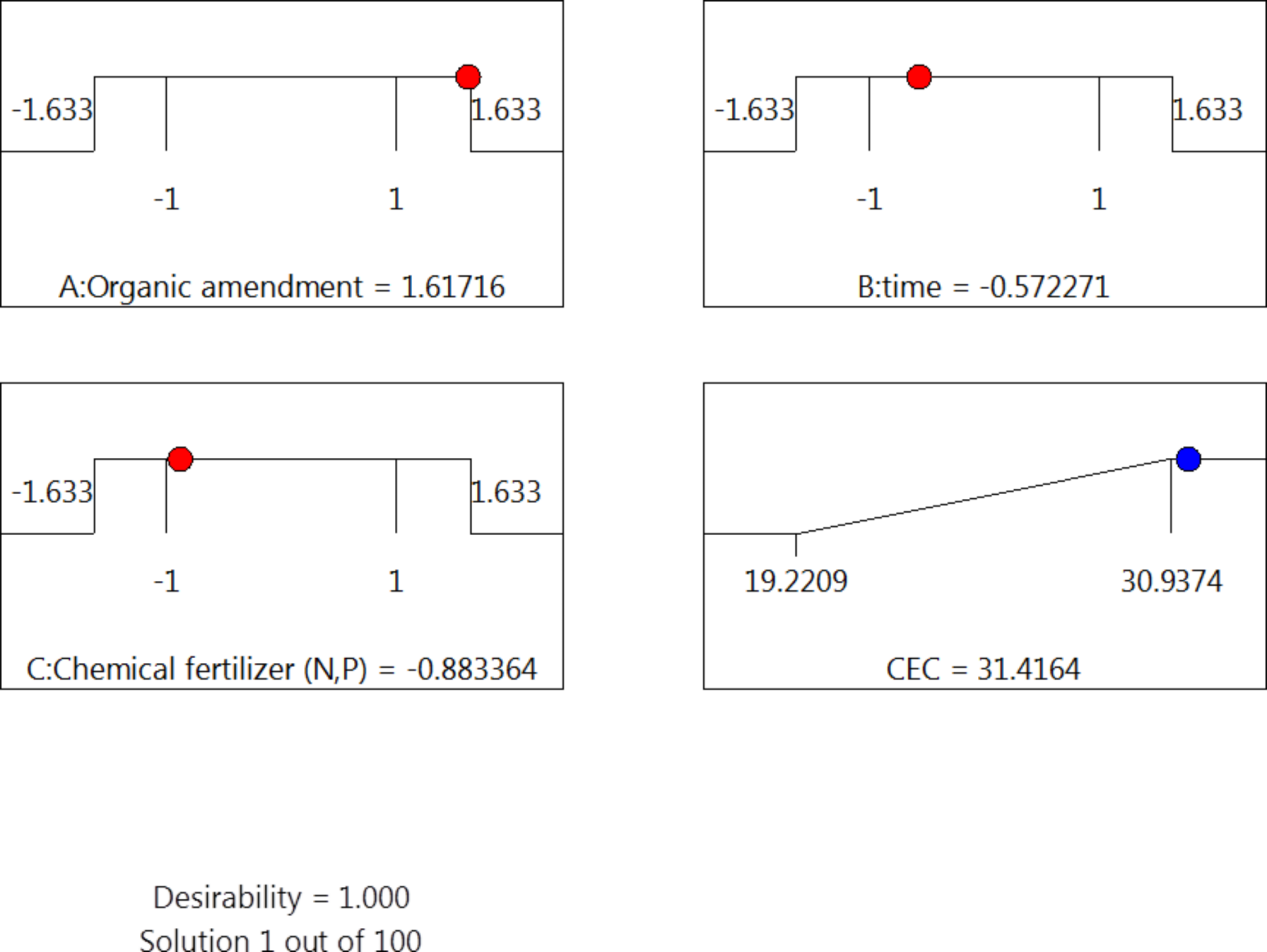
Schematic diagram illustrating the predected and acual values of of keratin hydrolysate (**A**), time (**B**), and chemical fertilizer (**C**).

As illustrated in Fig. 5, the use of 3D response surface and contour graphs showed that higher levels of CEC were reached with higher feather hydrolysate, lower amount of chemical fertilizer, and lower time. This result suggested that feather hydrolysate can be a more effective and sustainable alternative to chemical fertilizers for increasing CEC in soil. To estimate the ideal point of variation within experimental limitations, a second-order polynomial function was fitted to the experimental data (non-linear optimization algorithm). With a predicted CEC of 31.416 cmol_c_ kg^−1^ (Fig. 6), the maximum point of the polynomial model presented the ideal values for the three variables under investigation: feather hydrolysate as an organic amendment (20.147 ml kg^−1^ soil), time (27 days), and N-P chemical fertilizer (42.3%).

Y_CEC_ = 24.03 + 2.99 A − 0.4554 B − 0.5500 C + 0.2422 AB − 0.9269 AC − 0.0284 BC + 0.6288 A^2^ − 0.4467 B^2^ − 1.00 C^2^.

The effect of feather keratin hydrolysates on the main chemical parameters of soil after harvest relative to untreated soil were illustrated in Table 6. There was no significant effect on soil pH even though keratin hydrolysates or chemical fertilizers treated soil compared to control, which were relatively close. Additionally, available K and P (mg kg^−1^) were the highest significant, which increased about 2.29 and 1.56 folds relative to control treatment and about 0.91 and 0.74 relative to recommended chemical fertilizer addition, respectively. Soil CEC was decreased related to its optimization value, while its value was still higher compared to control (1.45 fold) and chemical fertilizer (1.34 fold). The obtained results indicate that the application of feather keratin hydrolysate to soil is highly effective in improving soil chemical characterizations and is suitable for plant nutrient availability.

**Table 6:** Some parameters of soil and maize plant grown in calcareous soil treated with recommended chemical fertilizer and feather keratin hydrolysates after 60 days of germination.

Treatment	Unit	*C	CF	K	LSD (0.05)	CV (%)
*Soil parameter*						
pH (1:2.5, W: V)	–	8.26 ± 0.05^a^	8.31 ± 0.08^a^	8.25 ± 0.01^a^	0.10^ns^	0.63
Avail. K	mg kg^−1^	244.12 ± 6.06^c^	558.60 ± 23.05^b^	614.12 ± 11.59^a^	30.57	3.24
Avail. P	mg kg^−1^	4.86 ± 0.33^b^	7.26 ± 0.84^ab^	9.27 ± 1.89^a^	2.41	16.92
CEC	cmol_c_ kg^−1^	14.93 ± 0.53^b^	16.15 ± 1.52^b^	21.65 ± 0.29^a^	1.89	5.39
OM	%	1.01 ± 0.03^c^	1.69 ± 0.11^b^	2.15 ± 0.03^a^	0.14	4.43
*Plant parameter*						
Shoot leave no.	–	6 ± 0.58^a^	7 ± 0.29^a^	7 ± 0.76^a^	1.15	8.52
Leave surface area	m^2^ gm^−1^	15.80 ± 3.29^c^	91.53 ± 14.80^b^	125.62 ± 9.29^a^	20.51	13.22
Shoot fresh weight	gm	12.20 ± 3.41^c^	84.55 ± 2.46^b^	113.78 ± 8.02^a^	10.45	7.45
Shoot dry weight	gm	4.80 ± 1.16^b^	19.15 ± 3.75^a^	22.74 ± 1.26^a^	4.75	15.29
Plant height	cm	42.83 ± 3.33^c^	80.08 ± 7.56 ^a^	62.00 ± 4.00^b^	10.59	8.60
Root volume	cm^3^	30.00 ± 6.00^c^	66.33 ± 11.59^b^	89.00 ± 6.00^a^	16.57	13.42
Root fresh weight	gm	18.18 ± 3.64^c^	40.20 ± 7.03^b^	63.51 ± 11.80^a^	16.39	20.18
Root dry weight	gm	3.20 ± 0.60^b^	7.01 ± 1.01^a^	8.39 ± 0.48^a^	1.47	11.86

*C control treatment; *CF* recommended chemical fertilizer; *K* keratin hydrolysates; *LSD* least significant difference; *ns* non-significant at *P*-value less than 5%; *CV (%)* coefficient of variance.

Means in rows with the same letter(s) are not significantly different at the *P*-value less than 5%.

In this recent study, using soil amendment of keratin hydrolysate (20 ml kg^−1^ soil) after 60 days of germination significantly enhanced the maize growth parameters (Table 6). Leave surface area (SA, m^2^ gm^−1^) elevated by 695% and 37% relative to the control and chemical fertilizer treatments, respectively. Compared to the control treatment, the root volume, root fresh weight, and root dry weight of the hydrolysate-treated plants were 2.97, 3.49, and 2.62 folds, respectively. Whereas shoot fresh weight, dry weight, and plant height were 9.32, 4.74, and 1.45 folds, respectively. Moreover, it is observed that keratin treatment application increased significantly all the investigated growth parameters compared to the recommended chemical fertilizer application except plant height. Notably, increasing root volume could improve the ability of nutrient absorption from the soil. It is reported that the application of keratin hydrolysate increases the membrane permeability and improves ion uptake from the soil^38,39^. It was concluded that feather hydrolysate application improved Bengal gram seed germination and growth in the pot trail. In the treated plants, there was a significant increase in root hair numbers on the lateral root surface and the root nodule numbers were three-fold higher^36^. Another result reported that feather hydrolysate amended soil increased the dry weight of leaves (33%) and roots (64%) after 60 days of lettuce germination compared to controls relative to urea fertilizer addition^9^.

For the purpose of determining the CEC under optimized and predicted optimal circumstances, a confirmation study was conducted to evaluate the quadratic polynomial’s correctness. This treatment was repeated in triplicate for 27 days. The obtained value of CEC was 26.59 cmol_c_ kg^−1^, which was remarkably close to the CEC value in the optimized conditions.

CEC is considered one of the most important chemical characteristics of agricultural soils and is a fundamental indicator of soil fertility and plant nutrient availability. However, it is reported that there are significant correlations between CEC and some soil characteristics, including pH, particle size distribution (sand, silt, and clay, total carbonate content, clay mineralogy, and clay content^40^. There is a substantial positive correlation between pH and CEC; rising soil pH values lead to an increase in the quantity of negative charges. Our results indicated that increasing soil CEC may be the dissociation of organic matter’s carboxylic functional groups in feather keratein hydrolysate. It is recognized that carboxylic functional groups have a positive charge at a low pH and a negative charge at a high pH^40,41^. It is reported that feather waste hydrolysate contained different amounts of amino acids including phenylalanine, valine, leucine, isoleucine serine, proline, and methionine^22,42^. Notably, amino acids as organic compounds contain amine (–NH_2_) and carboxyl (-COOH) functional groups, along with a unique side chain (R group) to each amino acid. These basic and acidic groups react to form one positive charge and one negative charge due to the internal salt formation. Depending on the isoelectric point of keratin (pH 4.9), the ionic bond between ammonium cations and carboxylic anions is weakened at very low and high pHs. Extremely alkaline conditions cause the amine groups to deprotonate these bonds, while extremely acidic conditions cause the carboxylic groups to protonate them^43^. The applied keratin hydrolysate was alkaline (pH 9.10, Table 4), thus the carboxylic functional groups have a negative charge. That means keratin hydrolysate is a promising material to improve soil CEC.

## Conclusion

Recycled poultry feathers are considered a great source of essential minerals and amino acids. Applying keratin hydrolysate as an organic fertilizer, enriched in carboxylic functional groups (in alkaline nature), in combination with nitrogen and phosphorus fertilizers at doses lower than the recommended amount for maize, enhanced soil cation exchange capacity (CEC). This improvement in CEC boosted nutrient retention and availability for plant uptake. As a result, the applying keratin hydrolysate as an organic amendment significantly improved maize growth parameters after 60 days of germination. The main chemical parameters of keratin hydrolysate-treated soil after harvest showed no notable change in soil pH. However, available potassium and phosphorus (mg kg^−1^) and CEC values were higher compared to the control treatment and recommended chemical fertilizer addition for maize growth. The obtained results indicated that the application of feather keratin hydrolysate to soil effectively improved soil chemical characterizations and enriched plant nutrient availability. Furthermore, it reduced the quantity of synthetic nitrogen and phosphorus fertilizers, thereby mitigating potential environmental harm. Despite these promising results, further investigation is necessary to evaluate its effect on the maize yield quality and quantity. Overall, the study suggests that utilizing feather keratin hydrolysate as an organic amendment can be an effective strategy for sustainable agriculture practices. Further research should be investigated to study the effect of the hydrolyzed feather and N-P fertilizers integration on soil organic matter content and its maintenance period in soil for a long time, subsequently the impact on plant yield.

## Data Availability

All data produced during this study are included in this published article.
